# Natural scene statistics relate to perceptual salience of second-, third-, and fourth-order spatial correlations

**DOI:** 10.1186/1471-2202-14-S1-P16

**Published:** 2013-07-08

**Authors:** Ann M Hermundstad, John Briguglio, Mary M Conte, Jonathan D Victor, Gasper Tkacik, Vijay Balasubramanian

**Affiliations:** 1 Department of Physics and Astronomy, University of Pennsylvania, Philadelphia, PA 19104, USA; 2 Weill Cornell Medical College, New York, NY 10065, USA; 3 Institute of Science and Technology Austria, Klosterneuburg, Austria

## 

The statistical regularities of natural scenes are a starting point for understanding the characteristics of early visual processing, e.g. the center-surround architecture of retinal ganglion cell receptive fields. Can this matching between natural signal statistics and neural processing mechanisms be extended beyond the sensory periphery? Our recent work [1] revealed that human sensitivity to fourth-order correlations in synthetic textures, known to arise in cortex, is closely related to the structure of fourth-order spatial correlations in natural scenes. This leads us to propose a specific organizing principle: The perceptual salience of visual textures increases with the variance, or unpredictability, of the corresponding correlations over the ensemble of natural scenes. To test this principle, we examined the statistical regularities of binarized natural images as characterized by correlations between adjacent pixels within a 2 × 2 square. Local binary textures can be described by four types of second-order correlations between pixels arranged in vertical (β_V_), horizontal (β_H_) and diagonal (β_\ _and β_/_) configurations, four types of third-order correlations between pixel triplets (θ_1,2,3,4_), and one type of fourth-order correlation between pixel quadruplets (α). We measured the values of these correlations in a large ensemble of image patches, and we compared the results to psychophysical experiments that measure human sensitivity to synthetic visual textures. Both the ordering and magnitude of natural image variances was found to match perceptual sensitivities to synthetic textures generated with the corresponding correlations (Figure 1a). Furthermore, the principal components of the full 9D space of image statistics match the principal components of the corresponding space of sensitivities (Figure 1b-e). These results suggest that central neural mechanisms are efficiently tuned to the higher-order statistics of natural scenes.

**Figure 1:**
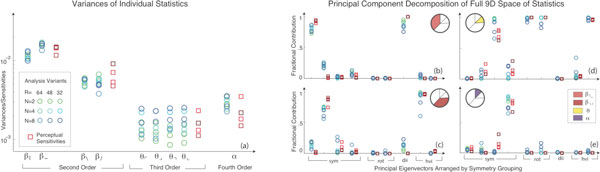
**(a) Variances in image statistics match perceptual sensitivities**. Principal components of image statistics match principal components of human sensitivities, as shown by the fractional contribution of **(b) **β_HV_, **(c) **β_/\_, **(d) **θ, and **(e) **α components to each principal eigenvector. Results are robust across image analyses and subjects.

## Methods

Natural Image Analysis: 88 grayscale images were block averaged in N × N patches to remove camera pixel matrix artifacts and divided into R × R patches. Image patches were whitened and binarized at their pixel intensity median. Psychophysical Analysis: Subjects were asked to identify a target (4-alternative forced choice) in brief (120-160 ms) presentations of 64 × 64-check binary images subtending 15 × 15deg. The target/background distinction was defined by a specific set of local image statistics, whose strengths were varied parametrically across trials [2].
